# A Chinese *Chan*-based Mind-Body Intervention Improves Memory of Older Adults

**DOI:** 10.3389/fnagi.2017.00190

**Published:** 2017-06-12

**Authors:** Agnes S. Chan, Winnie K. Cheung, Michael K. Yeung, Jean Woo, Timothy Kwok, David H. K. Shum, Ruby Yu, Mei-chun Cheung

**Affiliations:** ^1^Department of Psychology, The Chinese University of Hong KongHong Kong, Hong Kong; ^2^Chanwuyi Research Center for Neuropsychological Well-Being, The Chinese University of Hong KongHong Kong, Hong Kong; ^3^Department of Medicine and Therapeutics, The Chinese University of Hong KongHong Kong, Hong Kong; ^4^School of Public Health, The Chinese University of Hong KongHong Kong, Hong Kong; ^5^Menzies Health Institute Queensland and School of Applied Psychology, Griffith University, Gold Coast Campus, SouthportQLD, Australia; ^6^Department of Social Work, The Chinese University of Hong KongHong Kong, Hong Kong

**Keywords:** memory, lifestyle intervention, older adult, subjective memory complaint, *Chan* practice, aging

## Abstract

There is growing interest in the adoption of lifestyle interventions to remediate age-related declines in memory functioning and physical and psychological health among older adults. This study aimed to investigate whether a Chinese *Chan*-based lifestyle intervention, the Dejian Mind-Body Intervention (DMBI), leads to positive benefits for memory functioning in older adults. Fifty-six adults aged 60 years or older with subjective memory complaints (SMC) were randomly assigned to receive the DMBI or a control intervention (i.e., a conventional memory intervention; MI) once a week for 10 weeks; 48 of the adults completed the intervention. Participants’ verbal and visual memory functioning before and after the intervention were compared. In addition, changes in the participants’ subjective feelings about their memory performance and physical and psychological health after the intervention were examined. The results showed that both the DMBI and MI resulted in significant improvements in both verbal and visual memory functioning and that the extent of the improvements was correlated with participants’ level of performance at baseline. In addition, compared to the MI group, the DMBI group had significantly greater improvements in subjective physical and psychological health after the intervention. In summary, the present findings support the potential of the DMBI as an alternative lifestyle intervention for improving memory functioning, subjective physical and psychological health of older adults with SMC.

## Introduction

Subjective memory complaints (SMC) are common among older adults. The presence of SMC is often related to increased psychological distress, reduced mental health well-being, lower quality of life, and increased healthcare costs (Mol et al., 2007; Waldorff et al., 2009; Ito et al., 2013; Steinberg et al., 2013). Annually, approximately 6.67% and 2.33% of older adults with SMC experience their condition progressing to mild cognitive impairment (MCI) and dementia, respectively (Mitchell et al., 2014). Given the rapid growth of the elderly population in many countries (United Nations Department of Economic and Social Affairs, 2002), and the link between SMC and the increased risks of pathological aging (Mitchell et al., 2014), there is a need to identify effective interventions to improve the memory functioning of older adults with SMC as early as possible.

Over the past decades, many memory interventions for older adults have been developed (Gross et al., 2012). Conventional memory interventions maintain and enhance the memory functioning of older adults by teaching them internal (rehearsal, categorization, association, visual imagery) and external (use of external memory aids such as a diary, people around them) mnemonic strategies that facilitate the encoding and retrieval of information (Gross et al., 2012). According to a recent meta-analysis (Gross et al., 2012), the effect sizes of memory interventions ranged from medium to large values, indicating positive results of memory interventions in optimizing the memory functioning of older adults. In addition, one study showed that older adults had improvements in verbal memory that lasted 6 months after learning mnemonic and problem-solving strategies compared to those in a no-treatment control condition (Tsai et al., 2008), suggesting the presence of a relatively long-lasting effect. Furthermore, some studies have found positive effects of memory interventions on improving older adults’ self-perception of memory capabilities and psychological wellness (Floyd and Scogin, 1997; Valentijn et al., 2005; Belleville et al., 2006). These findings confirm the potential of memory interventions as an effective tool for enhancing memory functioning and psychological health in healthy older adults.

In addition to memory interventions, an emerging line of research is the use of lifestyle interventions to improve the memory functioning and wellness of older adults given the effectiveness and ease of accessibility of these interventions (Chan et al., 2005, 2014; Witte et al., 2009; Krikorian et al., 2010; Ruscheweyh et al., 2011; Clark et al., 2012; Yu et al., 2014). Generally, lifestyle interventions comprise an exercise component, a diet component, and at least one other component (e.g., counseling, smoking cessation, behavior modification) that aim to reverse pathology and/or delay disease progression by modifying high-risk health habits (Lifestyle Medicine, 2009). Lifestyle interventions, such as weight reduction and exercise interventions, and lifestyle-based occupational therapy interventions have been proven to be effective in slowing health decline and preventing loss of independence in older adults (Clark et al., 2012; Anton et al., 2013; Starr et al., 2014). Although considerable effort has been devoted to determining the effectiveness of lifestyle interventions in improving the wellness of older adults, little research has examined their effect on enhanced memory functioning. Some exceptions include findings on the beneficial effects of a 3-month caloric restriction intervention and a 12-week blueberry supplementation intervention on verbal memory performance in healthy older adults (Witte et al., 2009; Krikorian et al., 2010) and a relationship between healthy lifestyle and better memory performance in older adults (Flöel et al., 2008). Nevertheless, a potentially effective lifestyle intervention that enhances both memory functioning and wellness in older adults with SMC has not yet been clearly identified.

Recently, our study (Chan et al., 2014) demonstrated a positive effect of a Chinese *Chan*-based lifestyle intervention, the Dejian Mind-Body Intervention (DMBI), on improving the memory functioning of older adults. This innovative lifestyle intervention adopts a holistic approach that incorporates four interconnected components: *Chan* practice (self-awareness and self-control), *Nei Gong* practice, diet modification, and clearing of the bodily orifices. Our study showed that after a 10-session DMBI program, older adults showed clinically significant and reliable improvement in verbal and visual memory functioning, particularly among those with poorer memory functioning at baseline. These findings provided preliminary support for the DMBI as an effective lifestyle intervention to attenuate declining memory functioning in older adults. In addition, we found positive effects of the DMBI on physical and psychological health. After participating in the DMBI, community-dwelling older adults showed reduced levels of self-perceived psychological stress and improved self-rated health (Yu et al., 2014), and patients with depression reported a reduction in overall depressive symptoms (Chan et al., 2012b, 2013a). Nevertheless, the two previous studies with older adults lacked a control group for comparison (Chan et al., 2014; Yu et al., 2014). Thus, it was uncertain whether the memory and subjective health improvements brought by the DMBI were comparable to those brought by other conventional, well-established interventions, and whether such improvements were specific to the DMBI.

Therefore, given the encouraging findings of the DMBI in our previous studies, the present study aimed to further examine this issue by directly comparing the effects of the DMBI with a conventional memory intervention. Another purpose of this study was to explore factors such as participants’ age, gender, educational level, intervention attendance, and baseline performance that may influence the treatment effects of the DMBI. Based on the previous results of the DMBI in healthy older adults (Chan et al., 2014; Yu et al., 2014), it was anticipated that older adults with SMC would demonstrate both improved memory performance and improved subjective physical and psychological health after participating in the DMBI. In addition, we expected the extent of their improvement to be at least equal to, if not greater than, the level after participation in the memory intervention.

## Materials and Methods

### Participants

Sixty-nine community-dwelling adults aged between 60 and 91 years were recruited from health and social centers in the New Territories East regions in Hong Kong and through newspaper advertisements. All participants had SMC, indicated by a score of 3 or more on the Chinese Memory Symptoms Scale (Lam et al., 2005). The exclusion criteria included the following: a history of head injury or neurological/psychiatric disorder; an inability to walk; and severe illness. After a baseline assessment, participants who showed at least one of the following were also excluded: (1) a score higher than 7 on the short form of the Chinese Geriatric Depression Scale (CGDS-SF; Lee et al., 1993; Liu et al., 1998); (2) a score higher than 15 (i.e., in the moderate or severe range) on the Beck Anxiety Inventory (BAI; Beck et al., 1988); and (3) a score higher than 112 on the Chinese version of the Mattis Dementia Rating Scale (CDRS; Chan et al., 2003). After screening, 56 non-demented and non-depressed participants were randomly divided into the Memory Intervention (MI; *n* = 28) group and the DMBI (*n* = 28) group (**Table 1**). Two participants from the MI group and five participants from the DMBI group dropped out during the intervention period. The chi-squared test with Yates’s continuity correction did not show a significant group difference in the attrition rate, χ^2^ = 0.65, *p* = 0.42. In addition, one participant from the DMBI group failed to participate in the post-intervention assessment due to a knee injury. Thus, the final sample for the statistical analysis consisted of 26 participants from the MI group and 22 from the DMBI group. The mean attendance rates (i.e., number of classes attended in proportion to the total number of classes) for the two groups were both 91% and comparable*, t*(46) = 0.06, *p* = 0.95.

**Table 1:** Demographic characteristics of the Memory Intervention (MI) and Dejian Mind-Body Intervention (DMBI) groups.

	Group							
	MI (*n* = 26)			DMBI (*n* = 22)				
Variables	*M*		*SD*	*M*		*SD*	*t/*χ^2^	*p*
Age (years)	69.50		6.89	68.34		4.42	0.71	0.48
Gender (male/female)		5/21			27/15		1.01	0.32
Handedness (right/left)ˆ		25/1			22/0		0.00	1.00
Education (years)	8.69		4.62	9.82		4.69	0.84	0.41
CDRS (raw total score) #	131.48		6.18	132.82		7.70	0.66	0.51
BAI	3.38		3.66	2.73		2.49	0.71	0.48
CGDS-SF	2.12		1.95	2.14		1.49	0.04	0.97

*CDRS = the Chinese version of the mattis dementia rating scale; BAI = beck anxiety inventory; CGDS-SF = the short form of the Chinese geriatric depression scale. ˆChi-squared test with Yates’s continuity correction was performed for distributions violating the sample size assumptions of the chi-squared test. #One missing datum in the MI group*.

### Procedure

All participants provided informed written consent prior to the study. Before and after the 10-session intervention, participants were individually assessed using two memory tests: the Hong Kong List Learning Test (HKLLT; Chan, 2006) and the Visual Reproduction subtest of the Wechsler Memory Scale-III (WMS-III-VR; Wechsler, 2005). The participants also completed a global cognition assessment using the CDRS (Chan et al., 2003), an interview using the CGDS-SF (Lee et al., 1993; Liu et al., 1998), and the Chinese version of the BAI (Beck et al., 1988). In addition, a questionnaire on participants’ self-assessed changes in subjective physical and psychological health and memory performance was administered at post-assessment. All assessments and interviews were conducted by trained research assistants. The study was conducted in accordance with the Helsinki Declaration of the World Medical Association Assembly. This study was approved by the Joint Chinese University of Hong Kong – New Territories East Cluster (CUHK-NTEC) Clinical Research Ethics Committee.

### Intervention

The structure and format of the two intervention groups were designed to parallel each other with respect to their duration (i.e., 1.5 h/session) and frequency of sessions (i.e., once a week), group size, teaching and learning elements, in-session sharing, and discussions. The two interventions were administered by two different therapists who both had over 10 years of clinical experience as clinical psychologists.

#### Dejian Mind-Body Intervention (DMBI)

The DMBI was developed based on *Chanwuyi* (i.e., the Chinese *Chan* tradition, martial arts, and the *Chan* medical principle). The aims of the DMBI are to alleviate psychological distress by understanding the root of problems in accordance with Buddhist philosophy, enhance physical health by modifying one’s diet to reduce the intake of food that generates excessive internal heat, facilitate *Qi* and blood circulation by practicing *Nei Gong* (i.e., mind-body exercises), and clear out bodily orifices (i.e., the openings of the body) (Chan et al., 2008, 2009, 2010, 2011a,b, 2012a,b,c, 2013a,b, 2014, 2015, Yu et al., 2014). Throughout the 10 training sessions, the participants were taught the fundamental principles and techniques of the DMBI, and their progress was closely monitored by the therapist. The intervention consisted of three specific components.

(1) *Chan* practice: The participants were guided to foster self-awareness and self-control of their unrealistic desires (e.g., greed, anger and obsession) that had negative impacts on their physical and psychological health. The participants learned to modify their thought processes and thereby alleviate excessive unrealistic desires.
(2) *Nei Gong* practice: The participants were taught mind-body exercises (i.e., *Nei Gong)* that comprised sets of breathing exercises and gentle and calm movements. The purpose of practicing *Nei Gong* regularly is to reduce stress, develop the flexibility of one’s limbs, improve the circulation of *Qi* and blood, and foster self-awareness and self-control of one’s mental state. The practice duration was not fixed. The participants were advised to practice the exercise until they felt warm and relaxed but not to the point of overexertion. An elaboration of the basic principles and demonstration of *Nei Gong* is provided on our website^1^ and in two published books (Chan, 2010, 2013).
(3) Dietary modification: The participants were encouraged to consume food daily that was good for their health but, according to the *Chan* principle, to avoid the intake of food (e.g., ginger, garlic, green onion, spicy foods, eggs, meat, and fish) that generates excessive internal heat, thus adversely affecting emotional and physical health.

#### Memory Intervention (MI)

The 10-session Memory Intervention (MI) adopted in the present study was derived from Troyer et al. (2008). It provided memory training to older adults and incorporated three specific components.

(1) Psychoeducation: The participants were introduced to the nature of normal aging, MCI and the associated risk of Alzheimer’s disease, and different types of memory.
(2) Mnemonics training: The participants were taught and practiced mnemonic strategies that are well established in the existing literature, including spaced retrieval of information (Landauer and Bjork, 1978; Schacter et al., 1985; McKitrick et al., 1992; Davis et al., 2001), semantic association (Butters et al., 1997; Troyer et al., 2006; Hudon et al., 2011), implementation intentions (Gollwitzer, 1999), and the use of external memory aids such as a memory book, a calendar, or a list of things to do (Sohlberg and Mateer, 1989; West, 1995; Kime et al., 1996).
(3) Progress monitoring: The participants completed at-home assignments by recording how their everyday behaviors changed during their daily application of the memory strategies. If they encountered any problems with their practice, they discussed these in class with the therapist. The therapist provided feedback to ensure the participants’ success in applying the memory strategies in real life.

### Materials

#### Memory Tests

Two memory tests, including the HKLLT and WMS-III-VR, were used to evaluate the verbal and visual memory functioning, respectively, of older adults. The HKLLT, a standardized verbal learning test (Chan, 2006), has been widely used in empirical studies in the Chinese population (Chan et al., 2000, 2014; Cheung et al., 2010). In each trial, a list of 16 Chinese words randomly represented in four semantic categories was read to each participant. There were three learning trials followed by two delayed-recall trials (10-min and 30-min delays) and a recognition task. In addition, the WMS-III-VR, a common test for visual memory (Wechsler, 2005), was administered. This test required participants to first learn five sets of geometric forms and then draw them from memory immediately after each of these geometric forms was presented sequentially for 10 s. They were asked to recall the geometric forms again after a 30-min delay. The delayed-recall trial was followed by a recognition trial.

#### Global Cognition Assessment

The CDRS, a locally validated test that was culturally adapted from the Mattis Dementia Rating Scale (Chan et al., 2003), was administered to estimate the global cognitive functioning of older adults. This scale had good reliability with internal consistency values ranging from 0.70 to 0.90 and satisfactory construct validity (Chan et al., 2003).

#### Physical and Psychological Health Interviews

The Chinese versions of the CGDS (Lee et al., 1993; Liu et al., 1998) and BAI (Beck et al., 1988) were used to measure the participants’ depressive mood and anxiety symptoms, respectively. In addition, participants were asked to fill out a questionnaire at post-assessment to assess their subjective impression of the pre-post changes in their physical health, psychological status, and memory performance. The items in the questionnaire were designed based on the major findings of our previous studies, in which DMBI improved older adults’ physical and psychological health in areas of physical fitness, gastrointestinal health, sleep quality, and self-rated health (Chan et al., 2014; Yu et al., 2014). The questionnaire comprised 7 questions (i.e., Overall Physical Health, Mood, Respiratory Function, Gastrointestinal Function, Muscle Flexibility, Overall Psychological Health, Sleep Quality, and Memory Performance) on a 7-point Likert scale ranging from -3 (worsened) to +3 (improved), with higher scores indicating higher levels of subjective improvement.

### Data Preprocessing and Analysis

Prior to the statistical analysis, Kolmogorov–Smirnov tests were performed to check the normality of the dependent variables. Because violations of normal distributions were not significant for most of the tests, *p*s > 0.05, parametric tests were used for all subsequent statistical analyses. In addition, none of the results significantly change when non-parametric tests were used instead for dependent variables that violated the assumption of normality. For non-categorical variables, the MI and DMBI groups were compared using independent sample *t*-tests. For categorical variables, the two groups were compared using chi-squared tests, and the Yates’s continuity correction was applied if more than 20% of the cells had expected counts less than 5. To examine the changes in verbal and visual memory performance after intervention, paired *t*-tests were performed. In addition, to examine whether there were any significant changes in perceived memory, physical health, and psychological status, one-sample *t*-tests were performed. Cohen’s *d*s were calculated to evaluate the effect sizes of the pre-post changes (Cohen, 1988), with *d* values of 0.2, 0.5, and 0.8 indicating small, medium, and large effect sizes, respectively.

In addition, to examine the reliability of the intervention effects on memory functioning, Reliable Change Indices (RCIs) were computed, with RCIs > 1.28 reflecting reliable changes of the pre-post difference scores (Wise, 2004). Finally, to examine the associations between the improvements in verbal and visual delayed recall and baseline performance, age, gender, level of education, and attendance rate, Pearson’s correlations (two-tailed) were calculated. The strength of the associations was evaluated based on the Hopkins (1997) criteria, with *r* values of 0.1, 0.3, and 0.5 indicating small, medium, and large effect sizes, respectively. All statistical analyses were performed using SPSS 22.0 software (IBM Corporation, Armonk, NY, United States of America). Because all statistical comparisons were planned in advance, the significance level was set at 0.05 for all tests.

## Results

The major objectives of the present study were to investigate the effects of the 12-week DMBI compared to a control intervention on objective memory performance and subjective memory functioning and health status in older adults. Potential factors that may moderate these effects were also examined. Here, we first report the results of the change in objective memory functioning after the intervention and their moderating factors in Section “Change in Memory Functioning after Intervention”. We then report the results of subjective impressions of changes in memory, physical health and psychological health after the intervention in Section “Differential Improvements in Recognition Performance”. It should be noted that the DMBI and MI groups did not significantly differ in age, gender, handedness, years of education, or levels of global functioning, depressive, or anxiety symptoms (**Table 1**), *p*s > 0.32.

### Change in Memory Functioning after Intervention

We examined the potential benefits of the MI and DMBI on both verbal and visual memory functioning by comparing the pre-post changes on the HKLLT and WMS-III-VR. Verbal memory was measured by the HKLLT. Paired *t*-tests showed that after the intervention, both the MI and DMBI groups had significant improvements in their total learning, *p* < 0.01 (**Table 2**). Similar to the learning trials, the number of words recalled in the 10-min and 30-min delay trials was approximately 30% more than the baseline level, *p*s < 0.001. In the recognition trial, both intervention groups had significantly higher discrimination scores, indicating overall better recognition performance, *p*s < 0.01.

**Table 2:** Pre-post changes of the raw scores of the memory functioning indices in the Memory Intervention (MI) and Dejian Mind-Body Intervention (DMBI) groups.

	Group												
	MI (*n* = 26)						DMBI (*n* = 22)					
	Pre	Post	Difference	*t*	*p*	*d*	Pre	Post	Difference	*T*	*p*	*d*
**HKLLT**					
Total learning	22.42 (6.13)	28.92 (6.57)	6.50 (5.69)	5.82	<0.001ˆ***	1.14	22.91 (6.14)	27.32 (5.33)	4.41 (4.37)	4.73	<0.001ˆ***	1.01
10-min delayed recall	7.42 (3.11)	10.31 (3.12)	2.89 (2.42)	6.07	<0.001ˆ***	1.19	6.95 (3.09)	9.23 (3.10)	2.27 (1.81)	4.74	<0.001ˆ***	1.23
30-min delayed recall	7.42 (3.36)	9.96 (2.91)	2.54 (2.01)	6.46	<0.001ˆ***	1.26	6.59 (3.03)	9.00 (3.84)	2.41 (2.61)	4.33	<0.001ˆ***	0.92
Recognition (discrimination score)	83.17 (13.78)	91.59 (9.83)	8.41 (10.89)	3.94	0.001ˆ**	0.77	75.28 (16.53)	85.51 (15.95)	10.23 (9.36)	5.13	<0.001ˆ***	1.09
**WMS-III-VR**					
Immediate recall	71.04 (9.48)	74.62 (12.79)	3.58 (12.23)	1.49	0.15	0.29	64.32 (13.44)	71.05 (13.71)	6.73 (13.01)	2.43	0.02ˆ*	0.52
Delayed recall	42.15 (17.18)	61.65 (21.12)	19.50 (17.94)	5.54	<0.001ˆ***	1.09	40.50 (19.25)	51.77 (20.43)	11.27 (17.83)	2.97	0.007ˆ**	0.63
Recognition	42.62 (2.95)	43.35 (2.83)	0.69 (2.19)	1.61	0.12	0.32	41.27 (2.71)	41.91 (3.15)	0.64 (3.50)	0.85	0.40	0.18

*HKLLT = Hong Kong list learning test; WMS-III-VR = visuospatial reproduction subtest of the Wechsler Memory Scale III. Standard deviations are in parentheses. ^∗^*p* < 0.05, ^∗∗^*p* < 0.01, ^∗∗∗^*p* < 0.001*.

Similar results were obtained in visual memory as measured by the WMS-III-VR. Paired *t*-tests showed significant improvement in immediate recall, *t*(21) = 2.43, *p* = 0.02, *d* = 0.52, and delayed recall, *t*(21) = 2.97, *p* = 0.007, *d* = 0.63, in the DMBI group. However, the MI group showed significant improvement only in delayed recall, *t*(25) = 5.43, *p* < 0.001, *d* = 1.09 (**Table 2**), but not in immediate recall, *p* > 0.05. The significant improvement in immediate recall was still detected only in the DMBI group even when the baseline level of performance was matched between the two intervention groups by removing the two participants who had the highest immediate recall scores at baseline in the MI group, *p* > 0.10.

To examine whether the intervention effects on memory functioning were reliable changes, RCIs were computed based on the pre-post difference scores on the 10-min delayed recall on the HKLLT and the delayed recall on the WMS-III-VR for each intervention group based on the criteria suggested by Wise (2004). We found that 45.45% of the participants from the MI group and 53.85% of the participants from the DMBI group showed reliable improvement in the 10-min delayed recall on the HKLLT. In addition, 50.00% of the participants from the MI group and 53.85% of the participants from the DMBI group showed reliable improvement in delayed recall on the WMS-III-VR. No significant group differences were found in the number of participants who demonstrated reliable increases in either of these delayed-recall variables [HKLLT: *x*^2^(1) = 0.34, *p* = 0.56; WMS-III-VR: *x*^2^(1) = 0.07, *p* = 0.79]. Thus, the improvement of verbal and visual memory after the intervention was reliable in approximately half of the participants from each intervention group, suggesting that the DMBI might be as effective as the MI at improving the memory functioning of older adults with SMC.

To examine whether the extent of improvement in memory functioning was related to the participants’ age, gender, level of education, attendance rate, or baseline performance, Pearson’s correlations were calculated. The results showed significant and negative correlations between the baseline performance on the 10-min delayed recall on the HKLLT and the delayed recall on the WMS-III-VR and the respective pre-post change of performance in the two tests after the intervention [HKLLT: *r*(47) = -0.34, *p* = 0.02; WMS-III-VR: *r*(48) = -0.31, *p* = 0.03]. This finding suggested that older adults with lower memory functioning levels at baseline might have had greater improvement in both verbal and visual memory functioning after the intervention compared to older adults who had higher memory functioning levels at baseline. In addition, there were no significant correlations among age, gender, level of education, attendance rate, and pre-post change of memory performance, *p*s > 0.05.

### Differential Improvements in Recognition Performance

It is noted that both the DMBI and MI groups had significant improvements in the recognition trial of the HKLLT, but not in the recognition trial of the WMS-III-VR (**Table 2**). We therefore examined if this discrepancy was resulted from (1) a ceiling effect in the recognition scores on the WMS-III-VR; or (2) a difference in the baseline performances between the recognition trials of the two memory tests, thus limiting the room for improvement in the recognition trial of the WMS-III-VR.

First, to examine the ceiling effects, the numbers of participants performing at the near-perfect level (defined by a score greater than 90% of the maximum score in the recognition trials) on the HKLLT and WMS-III-VR at baseline were compared in the DMBI and MI groups separately. Chi-squared tests showed that there were no significant differences between the HKLLT and WMS-III-VR in the number of participants performing at the near-perfect level at baseline in either of the groups (DMBI: χ^2^ = 0.90, *p* = 0.34; MI: χ^2^ = 0.09, *p* = 0.76). Therefore, the different findings of improvements could not be attributed to a greater ceiling effect on the WMS-III-VR. Second, to examine the baseline performances, *Z* scores in the recognition trials at baseline were compared between the HKLLT and WMS-III-VR in the DMBI and the MI groups separately. Paired *t*-tests showed that there were no significant differences between the two *Z* scores in either the DMBI (HKLLT: *M* = 0.19, *SD* = 0.78; WMS-III-VR: *M* = 0.39, *SD* = 0.62) or the MI group (HKLLT: *M* = 0.64, *SD* = 0.62; WMS-III-VR: *M* = 0.68, *SD* = 0.79), *p*s > 0.35, suggesting that the discrepancy in improvement of recognizing verbal and visual stimuli could not be attributed to a better baseline performance on the WMS-III-VR either.

### Subjective Impression of Change of Memory, Physical Health, and Psychological Health after Intervention

**FIGURE 1:**
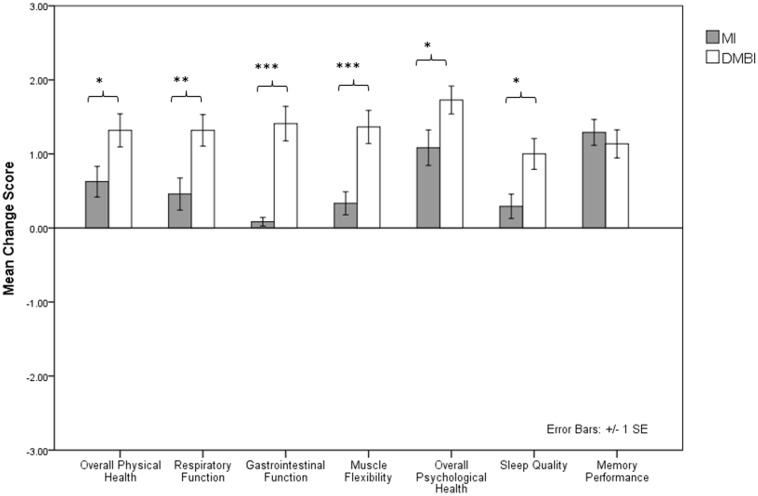
Mean improvement score of seven indices indicating changes in subjective physical and psychological health and memory performance (i.e., Overall Physical Health, Respiratory Function, Gastrointestinal Function, Muscle Flexibility, Overall Psychological Health, Sleep Quality, and Memory Performance) in the Memory Intervention (MI; *n* = 24) and Dejian Mind-Body Intervention (DMBI; *n* = 22) groups. ^∗^*p* < 0.05, ^∗∗^*p* < 0.01, ^∗∗∗^*p* < 0.001.

We also examined the benefits of the MI and DMBI on participants’ subjective impressions of their pre-post changes in memory functioning and physical and psychological health. One-sample *t*-tests showed significant differences on seven indices, indicating changes in subjective physical and psychological health and memory performance in the DMBI group [Overall Physical Health: *t*(21) = 5.94, *p* < 0.001; Respiratory Function: *t*(21) = 6.22, *p* < 0.001; Gastrointestinal Function: *t*(21) = 6.02, *p* < 0.001; Muscle Flexibility: *t*(21) = 6.10, *p* < 0.001; Overall Psychological Health: *t*(21) = 9.18, *p* < 0.001; Sleep Quality: *t*(21) = 4.81, *p* < 0.001; Memory Performance: *t*(21) = 6.00, *p* < 0.001] (**Figure 1**). In addition, the MI group showed significant improvements in five of these indices [Overall Physical Health: *t*(23) = 3.02, *p* = 0.006; Respiratory Function: *t*(23) = 2.11, *p* = 0.05; Muscle Flexibility: *t*(23) = 2.15, *p* = 0.04; Overall Psychological Health: *t*(23) = 4.51, *p* < 0.001; Memory Performance: *t*(23) = 7.37, *p* < 0.001]. However, independent sample *t*-tests showed significant group differences in the improved scores for Overall Physical Health, *t*(44) = -2.28, *p* = 0.03, Respiratory Function, *t*(44) = -2.83, *p* = 0.007, Muscle Flexibility, *t*(38.08) = -3.78, *p* = 0.001, and Overall Psychological Health, *t*(44) = -2.08, *p* = 0.04, but not for Memory Performance, *p* > 0.05. That is, compared to the MI group, the DMBI group had significantly greater improvements in subjective physical and psychological health after the intervention.

## Discussion

The present study examined the effectiveness of the DMBI, a Chinese *Chan*-based lifestyle intervention, at improving the memory functioning of older adults with SMC. The results showed that the DMBI, similar to a well-established mnemonic memory intervention, had positive effects on the improvement of memory functioning of older adults with SMC. Specifically, after a 10-session intervention, older adults demonstrated significant improvement in immediate- and delayed-recall trials in both the verbal and visual memory tasks. In addition, the extent of verbal and visual memory improvement was found to be correlated with the level of performance at baseline. That is, older adults with worse performance at baseline had a larger benefit on their memory functioning after the intervention. Overall, the current findings are consistent with the notion that cognition is plastic even in older age (Verhaeghen et al., 1992; Singer et al., 2003; Rebok et al., 2007; Willis and Schaie, 2009). Hence, appropriate interventions could improve the memory functioning of older adults with SMC, even those with comparatively poor memory at baseline.

The present study also demonstrated that the memory-enhancing effect of the 10-session DMBI was slightly greater than that of other lifestyle interventions. Whereas previous studies showed that a 3-month caloric restriction program induced a 20% increase in delayed recall (Witte et al., 2009) and a 6-month low-intensity aerobic exercise training induced a 10% increase in immediate recall (Ruscheweyh et al., 2011), the present findings demonstrated an approximately 30% improvement in both verbal and visual memory. In addition, the memory improvements in the DMBI were unlikely to be solely attributed to practice effects. That is, according to the WMS-III technical manual (Wechsler, 2005), adults between 55 and 89 years old gained average scaled scores of 1.4 in both the immediate and delayed recall trials of WMS-III-VR at a retesting that took place 2–12 weeks (*M* = 5.1 weeks) after the first testing. Whilst in the present study, the DMBI group gained average scaled scores of 1.7 and 1.8 (i.e., equivalent to gains of 6.7 and 11.3 raw scores) in the immediate and delayed recall trials, respectively, after the 10-session intervention. With a longer test-retest interval in the present study, the influence of practice effect was expected to be less pronounced than that reported in the manual. Furthermore, the degree of verbal memory improvement on retesting due to practice effects is uncertain, yet such improvement was less likely to be solely attributed to the practice effects either, given that the DMBI group had greater improvements in recall of verbal stimuli than visual stimuli in general. More importantly, the extent of objective and subjective memory improvement found in older adults who received the DMBI was comparable to those who participated in the MI, suggesting that the DMBI can be considered an alternative treatment for older adults with SMC.

The present findings are consistent with those reported in two of our previous studies that presented preliminary data on the benefits of the DMBI for improving memory functioning and self-rated health and reducing self-perceived psychological stress in older adults (Chan et al., 2014; Yu et al., 2014). The present study further verified the benefits of the DMBI for memory functioning and the subjective physical and psychological health of older adults with SMC, and it showed that these benefits were at least comparable to a conventional, well-established memory intervention. Although the DMBI does not involve explicit learning of mnemonic strategies or direct cognitive stimulation, studies consistently show that the DMBI is effective at improving the memory functioning of older adults and other clinical populations (Chan et al., 2014, 2011b, 2015). These beneficial effects may be due to a distinctive characteristic of this intervention. The DMBI fosters self-awareness and self-control of individuals’ unrealistic desires by engaging them in a thought modification process. Through this process, older adults recognize forgetfulness as a normal part of aging and realize that they would have unrealistic desires if they did not accept the fact that it takes them longer to learn new things and cannot remember information as well as they did before. More importantly, the necessity of seeking help for their memory complaints is brought into conscious awareness. Given that negative attitudes toward one’s memory performance are related to perceived forgetfulness in older adults (Mol et al., 2008), the approach of the DMBI, which facilitates the reduction of unrealistic desires, may explain the improvements found in the older adults in the DMBI group. We also found that compared to the MI group, the DMBI group had greater improvement in subjective physical and psychological health for older adults with SMC. This may be due to the “doing it naturally” approach adopted in the DMBI. That is, rather than placing strict regulations on diet modification and the duration and precision of their practice of mind-body exercises, older adults were provided with recommendations on how to abstain from food that generated excessive internal heat based on their eating habits and how to practice the mind-body exercises in response to their bodily signals. Another possible reason may be that the DMBI involves the practice of mind-body exercises that were developed based on the principles of Chinese *Chan* and Buddhism, which emphasize fostering a peaceful mind through the exercises to relieve psychological distress. Lastly, the DMBI may be a well-accepted intervention for older adults with SMC because they do not have to engage in the more effortful knowledge acquisition process of mnemonic strategies involved in the MI.

Despite the encouraging findings of the DMBI for improving the memory functioning and subjective physical and psychological health of older adults with SMC, there are several limitations in the present study. The study provides evidence of the short-term effects of a 10-session DMBI in a relatively small sample size of older adults. Further studies with a larger sample size and longitudinal follow-up are needed to expand our current knowledge on its long-term benefits for a wider population. The present study identifies participants’ baseline performance as the only potential factor that influences the memory-enhancing effects of the DMBI. To determine whether any other potential factors influence the intervention effects of the DMBI, training-related factors, such as the degree of concurrent training (Kwok et al., 2013) and the level of compliance with the training, could be examined in future studies. In addition, although the results showed that participants in the DMBI group might benefit verbal memory more than visual memory, the present study assessed participants’ memory functioning by using only one verbal and one visual memory test. Thus, further studies that include a diversity of verbal and visual memory tests may help to better evaluate the diverse range of improvements in verbal and visual memory resulted from the DMBI. Furthermore, the present study was conducted with a Chinese population; therefore, it remains unclear whether this intervention can be applied to non-Chinese population. All of these issues require further investigation. Despite these limitations, practicing the components of the DMBI is non-demanding, clearly comprehensible and highly applicable to the daily home lives of older adults, suggesting the potential of this *Chan*-based mind-body intervention as an efficient lifestyle intervention for older adults with SMC.

## Author Contributions

AC, JW, DS, TK, and RY contributed to the conceptualization of the study. WC and RY contributed to subject recruitment and data collection, AC, WC, and MY contributed to data analysis and initial draft of the manuscript. All authors contributed significantly to the later versions of the manuscript and approved the final version of the manuscript.

## Conflict of Interest Statement

The authors declare that the research was conducted in the absence of any commercial or financial relationships that could be construed as a potential conflict of interest.

## Footnotes

1 https://www.psy.cuhk.edu.hk/research/research-ctr/cwyctr/home

## References

[B1] Anton S. D. Karabetian C. Naugle K. Buford T. W. (2013). Obesity and diabetes as accelerators of functional decline: can lifestyle interventions maintain functional status in high risk older adults? *Exp. Gerontol.* 48 888–897. 10.1016/j.exger.2013.06.007 23832077 PMC3817488

[B2] Beck A. T. Epstein N. Brown G. Steer R. A. (1988). An inventory for measuring clinical anxiety: psychometric properties. *J. Consult. Clin. Psychol.* 56 893–897. 10.1037/0022-006X.56.6.893 3204199

[B3] Belleville S. Gilbert B. Fontaine F. Gagnon L. Mnard D. Gauthier S. (2006). Improvement of episodic memory in persons with mild cognitive impairment and healthy older adults: evidence from a cognitive intervention program. *Dement. Geriatr. Cogn. Disord.* 22 486–499. 10.1159/000096316 17050952

[B4] Butters M. A. Soety E. Becker J. T. (1997). “Memory rehabilitation,” in *Handbook of Neuropsychology and Aging*, ed. Nussbaum P. D. (Berlin: Springer), 515–527. 10.1007/978-1-4899-1857-4_34

[B5] Chan A. S. (2006). *Hong Kong List Learning Test*, 2nd Edn. Hong Kong: Department of Psychological and Integrative Neuropsychological Rehabilitation Center.

[B6] Chan A. S. (2010). *The Shaolin Chanwuyi: A Chinese Chan Buddhism*. Hong Kong: Chanwuyi Publishing.

[B7] Chan A. S. (2013). *Contemporary Application of Shaolin Medicine: Dejian Mind-Body Intervention*, 5th Edn. Hong Kong: Chanwuyi Publishing.

[B8] Chan A. S. Cheung M. C. Sze S. L. Leung W. W. Shi D. (2010). Shaolin Dan Tian breathing fosters relaxed and attentive mind: a randomized controlled neuro-electrophysiological study. *Evid. Based Complement. Alternat. Med.* 2011:180704 10.1155/2011/180704 PMC2957109 20976126

[B9] Chan A. S. Cheung M. C. Tsui W. J. Sze S. L. Shi D. (2011a). Dejian mind-body intervention on depressive mood of community-dwelling adults: a randomized controlled trial. *Evid. Based Complement. Alternat. Med.* 2011:473961 10.1093/ecam/nep043 PMC3136532 19474241

[B10] Chan A. S. Choi A. Chiu H. Lam L. (2003). Clinical validity of the Chinese version of Mattis Dementia Rating Scale in differentiating dementia of Alzheimer’s type in Hong Kong. *J. Int. Neuropsychol. Soc.* 9 45–55. 10.1017/S1355617703910058 12570357

[B11] Chan A. S. Han Y. M. Sze S. L. Lau E. M. (2015). Neuroenhancement of memory for children with autism by a mind-body exercise. *Front. Psychol.* 6:1893 10.3389/fpsyg.2015.01893 PMC4676196 26696946

[B12] Chan A. S. Han Y. M. Sze S. L. Wong Q. Y. Cheung M. C. (2013a). A randomized controlled neurophysiological study of a Chinese Chan-based mind-body intervention in patients with major depressive disorder. *Evid. Based Complement. Alternat. Med.* 2013:812096 10.1155/2013/812096 PMC3892748 24489591

[B13] Chan A. S. Ho Y. Cheung M. C. Albert M. S. Chiu H. F. Lam L. C. (2005). Association between mind-body and cardiovascular exercises and memory in older adults. *J. Am. Geriatr. Soc.* 53 1754–1760. 10.1111/j.1532-5415.2005.53513.x 16181176

[B14] Chan A. S. Kwok I. C. Chiu H. Lam L. Pang A. Chow L. (2000). Memory and organizational strategies in chronic and acute schizophrenic patients. *Schizophr. Res.* 41 431–445. 10.1016/S0920-9964(99)00078-X 10728720

[B15] Chan A. S. Sze S. L. Cheung M. C. Han Y. M. Leung W. W. Shi D. (2011b). Dejian mind-body intervention improves the cognitive functions of a child with autism. *Evid. Based Complement. Alternat. Med.* 2011:549254 10.1155/2011/549254 PMC3092624 21584249

[B16] Chan A. S. Sze S. L. Cheung M. C. Lam J. M. Shi D. (2009). Dejian mind-body intervention improves the functioning of a patient with chronic epilepsy: a case report. *Cases J.* 2:9080 10.1186/1757-1626-2-9080 PMC2803877 20062717

[B17] Chan A. S. Sze S. L. Han Y. M. Cheung M. C. (2012a). A Chan dietary intervention enhances executive functions and anterior cingulate activity in autism spectrum disorders: a randomized controlled trial. *Evid. Based Complement. Alternat. Med.* 2012:262136 10.1155/2012/262136 PMC3359807 22666288

[B18] Chan A. S. Sze S. L. Siu N. Y. Lau E. M. Cheung M. C. (2013b). A Chinese mind-body exercise improves self-control of children with autism: a randomized controlled trial. *PLoS ONE* 8:e68184 10.1371/journal.pone.0068184 PMC3707921 23874533

[B19] Chan A. S. Sze S. L. Shi D. (2008). Traditional Chinese mind body exercises improve self control ability of an adolescent with Asperger’s disorder. *J. Psychol. Chin. Soc.* 9 225–239.

[B20] Chan A. S. Sze S. L. Woo J. Yu R. H. (2014). A Chinese Chan-based lifestyle intervention improves memory of older adults. *Front. Aging Neurosci.* 6:50 10.3389/fnagi.2014.00050 PMC3972479 24723885

[B21] Chan A. S. Wong Q. Y. Sze S. L. Kwong P. P. Han Y. M. Cheung M. C. (2012b). A Chinese Chan-based mind–body intervention for patients with depression. *J. Affect. Disord.* 142 283–289. 10.1016/j.jad.2012.05.018 22840618

[B22] Chan A. S. Wong Q. Y. Sze S. L. Kwong P. P. Han Y. M. Cheung M. C. (2012c). A Chinese Chan-based mind-body intervention improves sleep on patients with depression: a randomized controlled trial. *ScientificWorldJournal* 2012:235206 10.1100/2012/235206 PMC3353275 22623888

[B23] Cheung M. C. Chan A. S. Sze S. L. Leung W. W. To C. Y. (2010). Verbal memory deficits in relation to organization strategy in high-and low-functioning autistic children. *Res. Autism Spectr. Disord.* 4 764–771. 10.1016/j.rasd.2010.02.004

[B24] Clark F. Jackson J. Carlson M. Chou C. Cherry B. J. Jordan-Marsh M. (2012). Effectiveness of a lifestyle intervention in promoting the well-being of independently living older people: results of the well elderly 2 randomised controlled trial. *J. Epidemiol. Commun Health* 66 782–790. 10.1136/jech.2009.099754 PMC3412049 21636614

[B25] Cohen J. (1988). *Statistical Power Analysis for the Behavioral Sciences*, 2nd Edn. Hillsdale, NJ: Lawrence Erlbaum.

[B26] Davis R. N. Massman P. J. Doody R. S. (2001). Cognitive intervention in Alzheimer disease: a randomized placebo-controlled study. *Alzheimer Dis. Assoc. Disord.* 15 1–9. 10.1097/00002093-200101000-00001 11236819

[B27] Flöel A. Witte A. V. Lohmann H. Wersching H. Ringelstein E. B. Berger K. (2008). Lifestyle and memory in the elderly. *Neuroepidemiology* 31 39–47. 10.1159/000137378 18535399

[B28] Floyd M. Scogin F. (1997). Effects of memory training on the subjective memory functioning and mental health of older adults: a meta-analysis. *Psychol. Aging* 12 150–161. 10.1037/0882-7974.12.1.150 9100276

[B29] Gollwitzer P. M. (1999). Implementation intentions: strong effects of simple plans. *Am. Psychol.* 54 493–503. 10.1037/0003-066X.54.7.493

[B30] Gross A. L. Parisi J. M. Spira A. P. Kueider A. M. Ko J. Y. Saczynski J. S. (2012). Memory training interventions for older adults: a meta-analysis. *Aging Ment. Health* 16 722–734. 10.1080/13607863.2012.667783 22423647 PMC3430800

[B31] Hopkins W. G. (1997). *New View of Statistics.* Available at: http://www.sportsci.org/resource/stats/effectmag.html [accessed November 3 2016].

[B32] Hudon C. Villeneuve S. Belleville S. (2011). The effect of semantic orientation at encoding on free-recall performance in amnestic mild cognitive impairment and probable Alzheimer’s disease. *J. Clin. Exp. Neuropsychol.* 33 631–638. 10.1080/13803395.2010.547663 21644138

[B33] Ito K. Inagaki H. Sugiyama M. Okamura T. Shimokado K. Awata S. (2013). Association between subjective memory complaints and mental health well-being in urban community-dwelling elderly in Japan. *Geriatr. Gerontol. Int.* 13 234–235. 10.1111/j.1447-0594.2012.00883.x 23286567

[B34] Kime S. K. Lamb D. G. Wilson B. A. (1996). Use of a comprehensive programme of external cueing to enhance procedural memory in a patient with dense amnesia. *Brain Inj.* 10 17–26. 10.1080/026990596124683 8680389

[B35] Krikorian R. Shidler M. D. Nash T. A. Kalt W. Vinqvist-Tymchuk M. R. Shukitt-Hale B. (2010). Blueberry supplementation improves memory in older adults. *J. Agric. Food Chem.* 58 3996–4000. 10.1021/jf9029332 20047325 PMC2850944

[B36] Kwok T. C. Bai X. Li J. C. Ho F. K. Lee T. (2013). Effectiveness of cognitive training in Chinese older people with subjective cognitive complaints: a randomized placebo-controlled trial. *Int. J. Geriatr. Psychiatry* 28 208–215. 10.1002/gps.3812 22528470

[B37] Lam L. C. Lui V. W. Tam C. W. Chiu H. F. (2005). Subjective memory complaints in Chinese subjects with mild cognitive impairment and early Alzheimer’s disease. *Int. J. Geriatr. Psychiatry* 20 876–882. 10.1002/gps.1370 16116581

[B38] Landauer T. K. Bjork R. A. (1978). Optimum rehearsal patterns and name learning. *Pract. Aspects Memory* 1 625–632.

[B39] Lee H. C. B. Chiu H. F. Kowk W. Y. Leung C. M. (1993). Chinese elderly and the GDS short form: a preliminary study. *Clin. Gerontol.* 14 37–42.

[B40] Lifestyle Medicine (2009). *Lifestyle Medicine - Evidence Review.* Available at: https://c.ymcdn.com/sites/acpm.site-ym.com/resource/resmgr/lmi-files/lifestylemedicine-literature.pdf [accessed November 3 2016].

[B41] Liu C. Lu C. Yu S. Yang Y. (1998). Correlations between scores on Chinese versions of long and short forms of the Geriatric Depression Scale among elderly Chinese. *Psychol. Rep.* 82 211–214. 10.2466/pr0.1998.82.1.211 9520556

[B42] McKitrick L. A. Camp C. J. Black F. W. (1992). Prospective memory intervention in Alzheimer’s disease. *J. Gerontol.* 47 337–343. 10.1093/geronj/47.5.P337 1512440

[B43] Mitchell A. J. Beaumont H. Ferguson D. Yadegarfar M. Stubbs B. (2014). Risk of dementia and mild cognitive impairment in older people with subjective memory complaints: meta-analysis. *Acta Psychiatr. Scand.* 130 439–451. 10.1111/acps.12336 25219393

[B44] Mol M. Carpay M. Ramakers I. Rozendaal N. Verhey F. Jolles J. (2007). The effect of perceived forgetfulness on quality of life in older adults; a qualitative review. *Int. J. Geriatr. Psychiatry* 22 393–400. 10.1002/gps.1686 17044138

[B45] Mol M. E. Ruiter R. A. Verhey F. R. Dijkstra J. Jolles J. (2008). A study into the psychosocial determinants of perceived forgetfulness: implications for future interventions. *Aging Ment. Health* 12 167–176. 10.1080/13607860801972503 18389396

[B46] Rebok G. W. Carlson M. C. Langbaum J. B. (2007). Training and maintaining memory abilities in healthy older adults: traditional and novel approaches. *J. Gerontol. B Psychol. Sci. Soc. Sci.* 62 53–61. 10.1093/geronb/62.special_issue_1.53 17565165

[B47] Ruscheweyh R. Willemer C. Krger K. Duning T. Warnecke T. Sommer J. (2011). Physical activity and memory functions: an interventional study. *Neurobiol. Aging* 32 1304–1319. 10.1016/j.neurobiolaging.2009.08.001 19716631

[B48] Schacter D. L. Rich S. A. Stampp M. S. (1985). Remediation of memory disorders: experimental evaluation of the spaced-retrieval technique. *J. Clin. Exp. Neuropsychol.* 7 79–96. 10.1080/01688638508401243 3980682

[B49] Singer T. Lindenberger U. Baltes P. B. (2003). Plasticity of memory for new learning in very old age: a story of major loss? *Psychol. Aging* 18 306–317. 10.1037/0882-7974.18.2.306 12825778

[B50] Sohlberg M. M. Mateer C. A. (1989). Training use of compensatory memory books: a three stage behavioral approach. *J. Clin. Exp. Neuropsychol.* 11 871–891. 10.1080/01688638908400941 2592528

[B51] Starr K. N. P. McDonald S. R. Bales C. W. (2014). Obesity and physical frailty in older adults: a scoping review of lifestyle intervention trials. *J. Am. Med. Dir. Assoc.* 15 240–250. 10.1016/j.jamda.2013.11.008 24445063 PMC4023554

[B52] Steinberg S. I. Negash S. Sammel M. D. Bogner H. Harel B. T. Livney M. G. (2013). Subjective memory complaints, cognitive performance, and psychological factors in healthy older adults. *Am. J. Alzheimers Dis. Other Demen.* 28 776–783. 10.1177/1533317513504817 24363073 PMC4617766

[B53] Troyer A. K. Hfliger A. Cadieux M. J. Craik F. I. (2006). Name and face learning in older adults: effects of level of processing, self-generation, and intention to learn. *J. Gerontol. B Psychol. Sci. Soc. Sci.* 61 67–74. 10.1093/geronb/61.2.P67 16497956

[B54] Troyer A. K. Murphy K. J. Anderson N. D. Moscovitch M. Craik F. I. (2008). Changing everyday memory behaviour in amnestic mild cognitive impairment: a randomised controlled trial. *Neuropsychol. Rehabil.* 18 65–88. 10.1080/09602010701409684 17943615

[B55] Tsai A. Y. Yang M. Lan C. Chen C. (2008). Evaluation of effect of cognitive intervention programs for the community-dwelling elderly with subjective memory complaints. *Int. J. Geriatr. Psychiatry* 23 1172–1174. 10.1002/gps.2050 18496884

[B56] United Nations Department of Economic and Social Affairs (2002). *World Population Ageing, 1950-2050.* Available at: http://www.un.org/esa/population/publications/worldageing19502050 [accessed November 3 2016].

[B57] Valentijn S. A. van Hooren S. A. Bosma H. Touw D. M. Jolles J. van Boxtel M. P. (2005). The effect of two types of memory training on subjective and objective memory performance in healthy individuals aged 55 years and older: A randomized controlled trial. *Patient Educ. Couns.* 57 106–114. 10.1016/j.pec.2004.05.002 15797159

[B58] Verhaeghen P. Marcoen A. Goossens L. (1992). Improving memory performance in the aged through mnemonic training: a meta-analytic study. *Psychol. Aging* 7 242–251. 10.1037/0882-7974.7.2.242 1535198

[B59] Waldorff F. B. Siersma V. Waldemar G. (2009). Association between subjective memory complaints and health care utilisation: a three-year follow up. *BMC Geriatr.* 9:43 10.1186/1471-2318-9-43 PMC2759924 19775441

[B60] Wechsler D. (2005). *Wechsler Memory Scale III (Chinese): Manual.* Taipei: Psychological Corporation.

[B61] West R. L. (1995). “Compensatory strategies for age-associated memory impairment,” in *Handbook of Memory Disorders*, eds Baddeley A. D. Wilson B. A. Watts F. N. (Oxford: John Wiley & Sons), 480–500.

[B62] Willis S. L. Schaie K. W. (2009). Cognitive training and plasticity: theoretical perspective and methodological consequences. *Restor. Neurol. Neurosci.* 27 375–389. 10.3233/RNN-2009-0527 19847065 PMC3607292

[B63] Wise E. A. (2004). Methods for analyzing psychotherapy outcomes: a review of clinical significance, reliable change, and recommendations for future directions. *J. Pers. Asses.* 82 50–59. 10.1207/s15327752jpa8201_10 14979834

[B64] Witte A. V. Fobker M. Gellner R. Knecht S. Flel A. (2009). Caloric restriction improves memory in elderly humans. *Proc. Natl. Acad. Sci. U.S.A.* 106 1255–1260. 10.1073/pnas.0808587106 19171901 PMC2633586

[B65] Yu R. Woo J. Chan A. S. Sze S. L. (2014). A Chinese Chan-based mind–body intervention improves psychological well-being and physical health of community-dwelling elderly: a pilot study. *Clin. Interv. Aging* 9 727–736. 10.2147/CIA.S59985 24790425 PMC4003151

